# Geometric Models of Speciation in Minimally Monophyletic Genera Using High-Resolution Phylogenetics

**DOI:** 10.3390/plants14040530

**Published:** 2025-02-09

**Authors:** Richard H. Zander

**Affiliations:** Missouri Botanical Garden, 4344 Shaw Blvd., St. Louis, MO 63110, USA; rzander@mobot.org

**Keywords:** fractals, hollow curves, meta-law in physics, rule of four, surfactant peripatric interface, Zipf’s law

## Abstract

High-resolution phylogenetics using both morphology and molecular data reveal surfactant-like trait buffering of peripatric descendant species that facilitate resilience for supra-specific entities across geologic time. Regular polygons inscribed in circles model balanced areas of survival of various numbers of new species in one genus. This model maximizes the peripatric survival of descendant species, with populations partly in allopatric habitats and in sympatric areas. It extends the theory advanced with Willis’s Age and Area hypothesis. Hollow curves of the areas bounded between a series of inscribed regular polygons and their containing circles show a ranked progression governed by similar power laws of other phenomena, including Zipf’s law and a universal meta-law in physics. This model matches best the physics meta-law (law of laws) but is only one of several somewhat different curves generated by somewhat different processes. A rule of four can explain why most genera in vascular plants exhibit a hollow curve of optimally one to five species per genus. It implies a constraint on variation that enhances survival and provides a physics explanation for the monophyletic skeleton of macrogenera. A high-resolution form of ancestor–descendant analysis is compared to traditional phylogenetic analysis to best modeling of the demonstrable results of evolutionary processes. Arguments are advanced for the preservation of scientific concepts of taxa over cladistic clades.

## 1. Introduction

The intent of this study is to investigate the fundamental reason why graphing species per genus and general per family always produce much the same hollow curve (“a power law”). This universal feature is connected with the observation that there are about five species in every minimally monophyletic group of one ancestral species and four immediate descendant species; while, in addition, robust species are generally limited to about four new traits per speciation event, leading to a generalized “rule of four”. This is a new science, and for important possibly unfamiliar terminology, see Glossary.

There are three methods of generating an evolution-based classification in the context of at least some fossil evidence: (1) *Evolutionary taxonomy*, which is the so-called “traditional” or standard, classical approach (often deprecated [1,2,3]). This method works with morphological data (where “morphology” includes all expressed traits) grouping species by overall similarity but emphasizing conservative traits, not neglecting homology assessment, and recognizing gaps between clusters of taxa as important. The product is a multi-chotomously branching cactus-like diagram or a dendrogram hybrid with inferred ancestral taxa at nodes ([4], p. 97). It generates hierarchic phyletic keys and supra-specific taxa of various sizes. Statistics are little used in practice. (2) *Cladistics* emphasizes “common ancestry”. It is a 50-year-old technology that recognizes groups based on shared trait changes of groups of species in a cluster analysis summarized by a dichotomous tree with all species (as operational taxonomic units) terminal on the branches. The product is commonly a rather large monophyletic supra-specific group of species as pattern clades named with standard classical nomenclature. Taxa in the evolutionary taxonomy sense are not recognized as such. Statistical support of molecular taxonomy is of first-order Markov chains in Bayesian analysis with flat priors. (3) *High-resolution phylogenetics* is a new science (see Glossary), in previous papers under the name macroevolutionary systematics, that preserves the achievements of evolutionary taxonomy (taxa) leveraged with modern methods of numerical taxonomy (Shannon information) and produces monophyletic evolutionary trees, in which ancestral species inferred from both morphology and molecular data match, and is supported by Turing sequential Bayesian analysis with informative conjugate priors. It uses descent with modification for recognizing minimum monophyletic groups as genera of one ancestral species and a few descendants, with ancestral species strung together to form a multichotomous diagram of bursts of speciation as a detailed monophyletic evolutionary skeleton. The method is a superset of cladistic methodology, but multichotomous trees replace cladograms, data come from both morphology and molecular “ancestral signatures”, and cladistically paraphyletic taxa are assigned to the nodes as inferred ancestors.

The main actor in high-resolution phylogenetics is the minimally monophyletic group, or microgenus, in previous papers, equally descriptive, called a monothetic genus. This is considered to have an essential role in preserving the resilience of a lineage across millions of years [5] and characteristically has one ancestor and usually one to four immediate descendant species. Why is four optimal? Willis [6] wrote a challenging analysis of consistency in the skewed distribution of data on the frequency of species in variously sized areas and numbers of species in a genus. The same distribution was evident in a large number of data sets and was a hollow curve with small numbers well-represented and large numbers trailing off asymptotically. He correlated this curve with the apparent age of a taxon and its area of distribution. His conclusions were discussed and to some extent challenged by reviewers of his book. Chamberlain [7], for instance, pointed out that the distributions, with an “enormous preponderance of small genera”, were clearly exponential or power law ([8], p. 362) in origin (using other terms). Gleason [9] pointed out that simple geographic distribution extensions over time comprised only a partial explanation of the phenomena. Watkins [10] advised that the Yule birth–death distribution seems adequate to account for the exponential curves and pointed out that the “ordinary difficulty of the systematist” was where to draw the line between genera. Pearson [11] advanced support for natural selection as a major factor. Ricklefs [12] has summarized the controversy. The present paper offers new science that may better explain the commonality of hollow curves in taxonomy, biogeography, and other fields, combining a geometric model with newly developed rules for species generation.

Since reading about Vavillov’s Rule of Homologous Variation, in which characteristic forms of within-species variability (“remarkable parallel”) may be expected in other species [13], inference of general laws reflecting deeply hidden processes in nature has been a lifelong pursuit. Here, two widely applicable generalities that help regularize apprehension of evolutionary patterns are summarized: the rule of four establishing descendant species that are soap-like partially soluble sympatric to their ancestral species and also partially soluble to new allopatric habitats, and a geometric interpretation of balanced peripatric speciation obeying a Zipf-like [14], ([15] p. 403) rule of rank progression limiting competition to one to four immediate descendant species. These generate ancestor–descendant trees that are strongly supported by calculations with morphological data on a multichotomous second-order Markov chain, using Turing–Shannon sequential Bayes as statistical support [16].

There have been many attempts to define a genus, classically by its “naturalness” or discontinuities [17]. The genus is here defined as the minimally monophyletic group (MMG), consisting of one ancestral species and a few (mostly one to four) immediate descendant species, and further characterized by the most recent set of evolved morphological traits being duplicated in each immediate descendant species, and the number of new traits in both ancestor and descendant is also optimally about four. It is somewhat like the phylogenetic species concept, which is the smallest group that shares a distinctive evolutionary history and also differs from other groups [18], but the MMG or microgenus has fractal features of self-similarity in numbers of descendants and traits across scales [19].

High-resolution phylogenetics is a modern extension of numerical taxonomy (also known as computational systematics, or automatic classification) in using mathematics and statistics to investigate evolutionary relationships. Cladistic parsimony is replaced by maximizing Shannon entropy, i.e., a decrease in uncertainty [20], while data sets are reduced to that for minimal monophyly, which minimizes trait convergence across taxa. Evolutionary skeletons are resolvable within larger monophyletic units by linking ancestral to ancestral species of small, minimally monophyletic genera. Second-order Markov chains together with sequential Shannon–Turing Bayesian analysis provide strong statistical support. Several recent publications describe and justify this new concept in detail [19,21,22]. This concept lends directly to a definition of species as “any reasonable definition of a species that lends itself to conceptual participation in the evolutionary mechanics of minimally monophyletic groups”.

**Figure 1:**
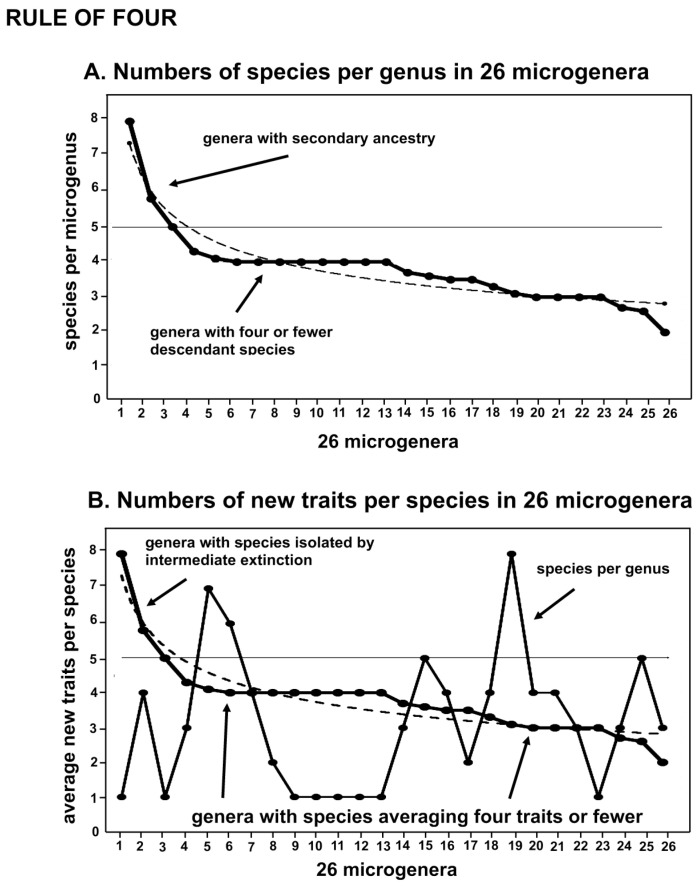
Species and traits per species. (**A**) Numbers of species each of 26 minimally monophyletic groups (microgenera). Genera with more than five species (one ancestor and four descendants) are few, and immediate descendants may have descendants of their own. The dotted line is an Excel regression trendline demonstrating that this is a power rule. (**B**) Average new traits per species (the novon set) in 26 microgenera. A few genera average many traits per species, explained by serial extinction. Again, the trendline demonstrates that this is a power rule distribution. The number of new traits per species (solid line) is independent of the number of species per genus (dotted line). The comparison of these graphs reveals a self-similarity of the constraint of information across rank hierarchies.

**Figure 2:**
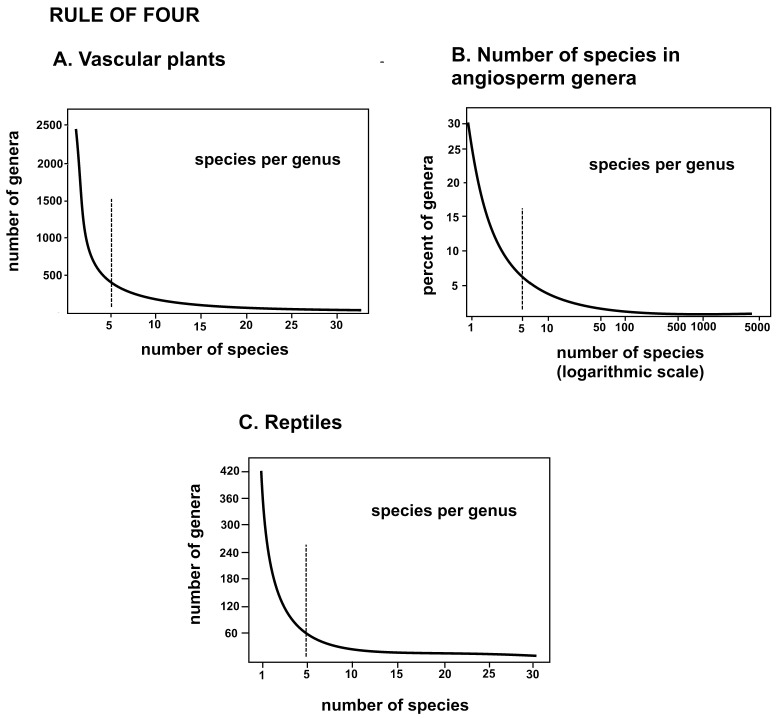
General evidence for the evolutionary rule of four (optimally four immediate descendants per ancestral species). Heavy lines plot the hollow curve. The dotted line at five species demonstrates a large number of species of one to five species. (**A**) All vascular plants, with a number of species cut off at 35. (**B**) Angiosperm genera, *X*-axis exponential to show the existence of huge genera. (**C**) Reptiles, implying a similar rule of four in animals. The five-species optimality is potentially explained by constraints on the geometric dispersal of survival information during peripatric speciation.

Investigation of fractal evolution [19] found that morphology-based microgenera follow a complexity-related “rule of four” in which there are optimally four descendant species per ancestor and four newly evolved traits per species. Most genera are composed of one to five species, which is demonstrated by the graphs in Figure 1 and Figure 2. The term “rule of four” comes from a study in inorganic chemistry [23] which found that many compounds were composed of four basic chemical units. Why four? The chemists were puzzled but asserted the reality of this feature.

The organic evolutionary context is that among ca. 26 microgenera studied [24], the optimal number of immediate descendants of any ancestral species is four, and the number of traits in the novon (the new traits of each descendant species) is four (Figure 1). There is variation due to extinction and simple chance, but most numbers of descendant species range from four to zero depending on the genus studied. The older the genus, the fewer there are species in the genus [22], although ancestral species are not particularly more vulnerable to extinction than their descendants.

A previous morphological study of 26 microgenera [24] in the moss families Pottiaceae and Streptotrichaceae found that the average number of new traits in each of the 82 species was 3.44, with a range from 8.00 to 2.00 average per species depending on the genus. The distribution (Figure 1B) is largely ca. 4 new traits per species, with a few species showing an average of 5 to 8 new traits, and a larger number tapering off to 2, which is the smallest allowed to distinguish a species. This may be interpreted evolutionarily as a few genera experimenting with a large range of species, and the remainder gradually eroding with continuing extinction. The figures also demonstrate that the number of species in a genus (the jagged solid thin line) does not affect the average number of traits per species.

There is also evidence of a rule of four in molecular systematics data. A study [24] of several published papers by various authors on molecular systematics of the Pottiaceae found 71 entries of molecularly multiracial species including 46 different species. The number of molecular races among the 46 multiracial species was 208. The average number of molecular races for the 46 species (i.e., the number of internal nodes interpreted as signaling different sequences) was 4.5 for both paraphyletic and non-paraphyletic species. The total number of paraphyletic instances (molecular races that are also paraphyletic) among the 71 entries was 29. The number of paraphyletic species was 22 or 0.4 of the 46 multiracial species. The number of different apophyletic species (those distal to a paraphyly) in all sequences was 79. The average number of apophyletic species per paraphyly was 3.6. A total of 133 nodes were maximally distant (on the cladogram) between paraphyletic exemplars. The average number of nodes between most-distant paraphyletic exemplars of the same species was 4.5 (133/29). This suggests that molecular variants of ancestral species together generate 3.6 to 4.5 descendant, apophyletic species (including secondary ancestry).

A geometric model of radiative evolution might provide an explanation. Area-related explanations for budding evolution have been provided for the peripatric origin and isolation of new species [25,26]. The relevant and instructive new analogy is soap, a surfactant molecule that is soluble in water at one, ionic end, and in oil at the other, non-ionic end. A species consists of about four traits contributed from the new traits of the ancestral species plus about four traits newly evolved from old and perhaps useless traits of the ancestor. The new traits explore survival opportunities allopatrically and the old traits ensure a toehold sympatric with the ancestral species. Here, sympatry is any co-existence in habitat or geographic distribution, not necessarily at the same time. Peripatry is the peripheral area between the occurrence of the generative species and allopatric habitats. Competition has been shown among peripatric species of insects [27] and mammals [28]. The question is as follows: How many descendant species can this peripatry hold, given mutualism and competition?

Paraphyly means ancestral, and paraphyly is essential information for investigating descent with modification. Paraphyly does not mean “not monophyletic” contra Nordal and Stedje [29]; it in fact is good evidence for descent with modification. What is descending? The apophyletic taxon of the same rank (species, genus, family). Previous work in NK analysis with a random Boolean model [19] found that the fractal nature of species studied (26 bryophyte genera in two families) increased the tolerance of species to competitive interactions, particularly between descendants. It was found that about half the species studied were easily modeled as direct ancestors of other species. Reiseberg and Brouillet [30] estimated that at least 50% of all plant species are generated through local geographic speciation and are therefore paraphyletic. Funk and Omland [31] found undeniable species-level paraphyly or polyphyly in 23% of more than 2000 species sampled. According to Aldous et al. ([32], p. 322), about 63% of extant species have still living ancestors. Frey [33] suggested that paraphyletic scenarios are common or even the rule, while Gurushidze [34] supported the idea that pseudoextinction (disappearance of a progenitor taxon after generation of daughter taxa) is rare.

In the previous morphological study of 26 microgenera, each of the optimally four immediate descendant species was given the ca. four newest traits of their ancestral species, while developing optimally four new traits as modifications of older, apparently less useful ancestral traits. The model of five total species resulting from one ancestral species generating four additional species could be described with the fractal dimension of 1.16, or log 5/log 4, matching the Pareto distribution of 80:20, or 80% of the total generated by 20% of the source. Because descendant species were partly composed, probably by natural selection, of a modicum of the newest traits of the ancestor, it was suggested [19] that competition when entering new habitats was much lessened in the new species, a conclusion supported by NK/random Boolean modeling. This paper examines a possible geometric causal explanation for this instance of an evolutionary rule of four.

Geometric modeling offers a possible method for the retention of successful adaptive traits by botanical genera and families across the 66 million years of modern flora as suggested by Zander [16,22,35].

## 2. Results

### 2.1. Model 1

Zander [19] studied 26 genera each consisting of one ancestral species and a few immediate descendants, with occasional secondary ancestry (a descendant generating another descendant). These were termed microgenera, being minimally monophyletic units comprising larger monophyletic groups, and taxonomically treated at the genus level. The 26 microgenera were ordered in numbers of species and of newly evolved traits (the set called the novon) and graphed (Figure 1). In Figure 1A, there is a step-wise fall-off of numbers of species, with three genera having more than five species (one ancestor and four descendants), but the remainder reflecting a possible effect of gradual extinction. Compressing the graphs of graphs laterally would produce a vaguely hollow curve, but the numbers of species and traits are so few as to disguise this. A horizontal line across the y-axis demonstrates that most microgenera have five or fewer species. Genera with fewer than five species may be due to extinction (the older species) or relative recency (the younger species), but the latter is less probable given an apparent burst style of speciation.

Figure 1B graphs the average number of newly evolved traits (the set is the novon) per species per microgenus in the same 26 microgenera. The traits are ranked in descendant numbers. Some microgenera have many average traits per species, while the majority have four or fewer average traits per species per genus. The graph slopes off gradually, such that the average number of traits per species is probably random but simply largely limited to four or fewer. The x-axis has a different order of microgenera than in Figure 1A, and the dotted line demonstrates that the size of the microgenera in a number of species is not correlated with the average number of traits per species.

The dashed lines in Figure 1A,B are trendline regression curves fitted with the Microsoft Excel spreadsheet. They are both power curves showing very long, robust tails.

### 2.2. Model 2

The results of Model 1 were compared to previous graphing of the numbers of species per genus in larger groups of organisms. These groups were of classical taxonomic generation, and were not devised as microgenera, but clearly show a large mass of genera with only one to five species; thus, most genera are potentially minimally monophyletic groups. The graph of vascular plants (Figure 2A) from Stevens [36] shows a distinctive hollow curve, artificially cut off at 30 species, while that of the angiosperm genera (Figure 2B) is more recent [37], and the log x-axis shows an asymptotic weight of very large genera that are surely not microgenera and are caused in part by cladistic lumping of minimally monophyletic genera into much larger genera. The graph of the reptiles (Figure 2C) from François [38] shows a hollow curve identical to that of the vascular plants. It is clear that further analysis of minimally monophyletic groups has the potential for clarifying internal features of ancestor–descent and trait evolution and for potentially reducing the size of massive macrogenera represented in Figure 2B.

### 2.3. Model 3

**Figure 3:**
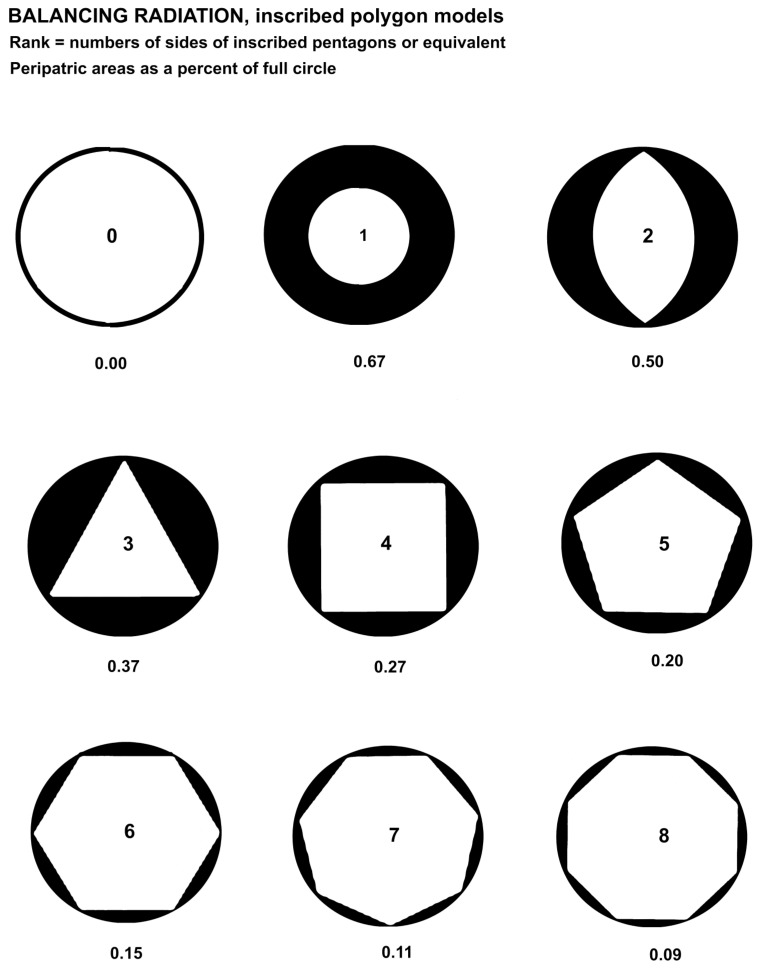
Regular polygons inscribed in circles modeling peripatric speciation and balanced competition between descendant species ranked 0 through 8 for a number of polygon sides. The black portion models the maximum habitat coverage of descendant species without inter-competition. Zero is just the ancestral species. One is a conjectural polygon of one side, the descendant balancing the ancestral species. Two is a conjectural polygon of 2 sides, balancing two descendant species. The remainder are actual regular polygons of 3 to 8 sides. Percent of the peripheral area cut off by the inscribed polygon or equivalent is evolutionarily important. Genera with fewer than six immediate descendant species may sustain less internal competition and have better chances of survival in the long term.

Peripatric speciation was modeled with regular polygons (Figure 3) inscribed in circles (Figure 3), with the area outside the polygon modeling, by analogy, newly evolved species with minimum conflict between each other. The peripatric descendant genera survive through partial adaptation to allopatric habitats while retaining adaptions to sympatric ancestral areas. This is theoretically due to retaining the most recent morphological traits of the ancestral species to balance about equal numbers of new traits. The formula for the area of the space between the inscribed regular polygon and the enclosing circle is given by Equation (1).

(1) $$\frac{Aoutside}{Acircle}=\frac{½nr^{2}-\sin\left(\frac{2\pi }{n}\right)}{\pi r^{2}}$$

The figures are ranked by number in Figure 3, and the black areas represent the maximal areas allotted by competition for each descendant. The empty circle represents the ancestral species. The third through eighth ranks are generated by Formula (1). Ranks one and two are mathematically absurd because of zeros (singularities) but are generated by Microsoft Excel trendlines, which may be expected to at least reflect the underlying natural processes. Most minimally monophyletic groups have at least a few descendants.

### 2.4. Model 4

**Figure 4:**
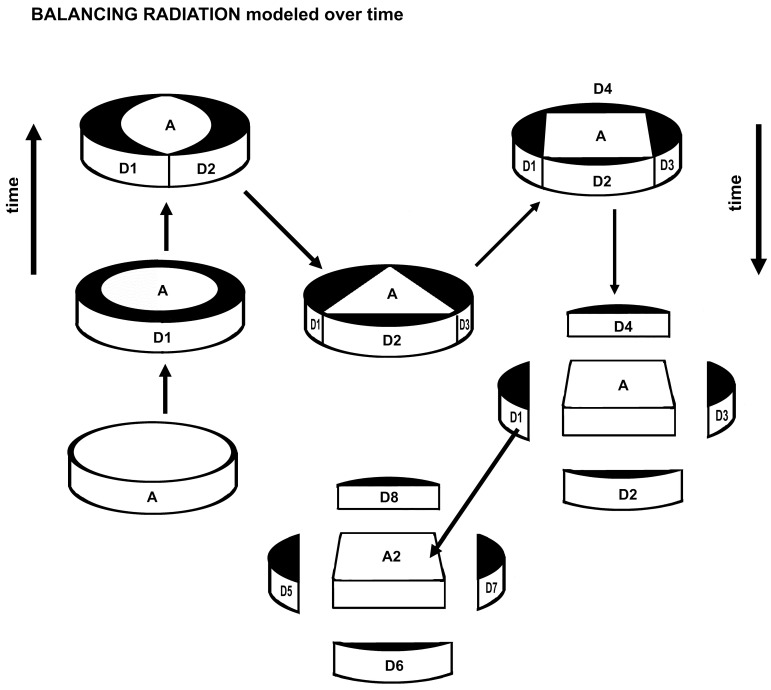
Model of peripatric speciation with increasing number of peripatric descendant species over time. Peripatric descendant species seldom originate beyond four in number, then disperse and eventually become ancestral species themselves. A = ancestral species, D = descendant species. This is a theoretical model of how competition might control numbers of less-than-successful immediate descendant species, which preserves successful traits in the lineage as a whole across geological time scales.

The various geometric forms (Figure 4) are arranged in the estimated order of generation of descendant species (D1 through D4) from one ancestral species (A). Speciation is modeled over time such that crowding through competition and/or the predictable decoherence of a system starved of energy by saturation leads to allopatric dispersal at about the four-sided model. The system is an evolutionary entity at a taxonomic rank higher than the species whose functionality arises from the mutuality of the ancestor–descendant relationship acting as a robust, flexible competitive agent through time. In the generation of one descendant (D1), the model assumes rapid advance to two descendants, then to three and four. This model punctuates equilibrium in extant species and focuses on immediate descendant species. Each descendant species may eventually have one or more further descendants after establishment allopatrically. The definition of a genus as a minimally monophyletic group consisting of one ancestral species and a few descendant species sharing the same newest ancestral traits is an exact definition but constituents vary with extinction and secondary ancestry. Of course, there are immediate descendant species with species of their own, and these may be assigned a different genus name if the fundamental feature of evolution, the elements involved in sharing the ancestral novon with the descendant species, is clearly changed, and there are indications of extant punctuated equilibrium (multiple descendants).

### 2.5. Model 5

**Figure 5:**
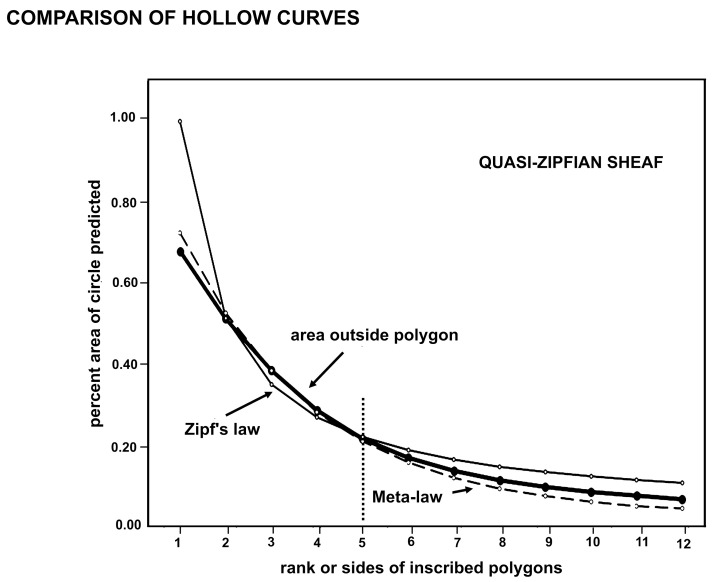
Hollow curves of power rules with negative exponents. The thick solid line is the area outside the regular polygon as a percent of the inscribed circle. The thin line is Zipf’s law showing polygons ranked by a number of sides as a harmonic series of fractions. The dotted line is Constantin et al.’s (2024) meta-law for a series of operators in physics formulae. These comprise a unit or sheaf of hollow curves, each apparently valid in describing closely associated aspects or contextual views of processes in nature. The physical law or joint cause is not and perhaps cannot be precisely modeled by the curves.

Geometric Equation (1) for the area outside an inscribed polygon generates results (Figure 5) that are quite like those of Zipf’s empirical law ([15], p. 403) and [39,40,41] that a list of measured values when sorted in decreasing order will exhibit the value of the nth entry as approximately inversely proportional to n. Zipf’s law is a power rule with the power of –1 (minus one), creating a harmonic series, and forms a linear line when plotted on a double logarithmic graph, otherwise a typical hollow curve, as in the thin line of Figure 5. Zipf’s Formula (2) indicates that the rank *r* of a progressive series approximates the inverse of the numbered rank with a power, *a*, with *a* approximating one.

(2) $$f\left(r\right)\sim \frac{1}{r^{a}}$$

A corollary of Zipf’s principle is Pareto’s principle, that is, 20% of a resource generates 80% of the total. This in turn has the Mandelbrot fractal dimension of 1.16 or log 5/log 4. This number was demonstrated [19] as being fractal or optimally four descendants arising from one ancestor resulting in a total of five species in each minimally monophyletic group (the microgenus).

In practice, Zipf’s law is not proven or exact when applied to everyday distributions, and a variant, the Zipf–Mandelbrot formula is described [42] as more accurate when numbers are small. Recently, Constantin et al. [42] have found that the frequency of process-label symbols in a large number of mathematical and physic formulae approximates Zipf’s law rather regularly, forming a similar and characteristic hollow curve. They found that a more exact match to their data was a simple exponential Formula (3) where, again, *r* is rank:

(3) $$f\left(r\right)\sim e^{-r/3}$$

Constantin et al. [42] suggested that this equation implies a heretofore hidden, universal, and powerful physical rule (hereafter the meta-law) operating across many disciplines and affecting all of perceivable nature [43]. Data for the calculation of the geometric areas by Equation (3) in Figure 5 are given in Table 1.

The ranked geometric models, Zipf’s law, and the meta-law formula, when graphed (Figure 5) together, form a sheaf of somewhat different hollow curves. The conjectural polygon of one side, the monogon, and that of two sides, the digon, are evaluated as values that are simply power law extensions of the remainder of the curve. The curves presented by Willis [6], p. 237, though many, are not clearly exponential or power law but are probably the latter as they trail off in a thick asymptote. Among them, however, is singled out the curve of genera with five species.

These values mapped in Figure 5 each create a clearly smooth curve, which matches closely the physics meta-law curve of Constantin et al. [42]. The meta-law curve is a better match than Zipf’s law for the geometric progression here suggested as a good model explaining the rule of four constraint of about one ancestral species having four immediate descendant species. The meta-law has the cachet of being dubbed a universal law by the developers, and the study suggests that the fractal evolution of these 26 genera of mosses is a real phenomenon.

Given that power law and exponential hollow curves need to be “fitted” to match data from empirical studies, one might conclude that there are many rules of four each fitting some hollow curve distinctive for particular processes in nature [44]. Processes that generate small numbers may be disconcertingly many [45], but most may involve competition for habitat or substrate. The number of petals in a flower is commonly three to five, perhaps through geometric physical constraint, while the number of stripes on the exposed skin of a snake is commonly five and may be traced to Turing patterning [46,47,48]. A single explanation may not account for the sheaf of hollow curves associated with small numbers generated by natural processes including those of evolution, but such may comprise a set of several simple processes. Not all the curves match the meta-law, and it is premature to lump the lot into one rule. Suffice that the sheaf of curves is “quasi-Zipfian”.

### 2.6. Model 6

**Figure 6:**
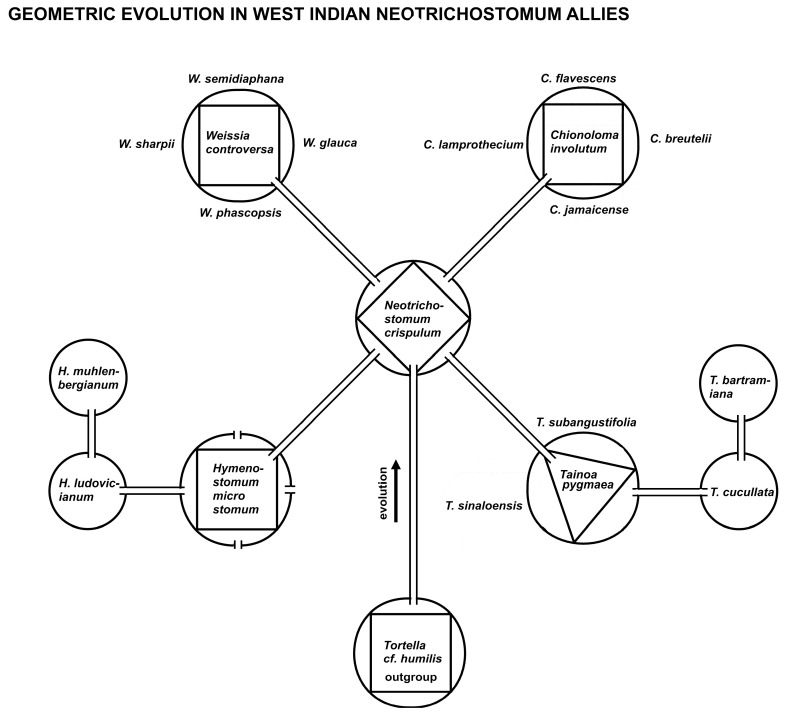
Evolutionary diagram of a group of moss species from West Indies and adjacent areas showing actual geometric rule-of-four descent with modification. Self-similarity is present at various genus and family levels, with four descendants of satellite genera and of the central ancestor of these genera. Two genera show secondary ancestry. Every ancestral species is supported by being the most generalist among the ingroup and also the most similar of all species to the outgroup (second-order Markov chain). Five species per genus is optimal and explains the hollow curves, and extinction trims away unsuccessful internally competing species of the genera.

A caulogram (ancestor–descendant stem-taxon tree) was fashioned from that of Zander [19], p. 41, of the “*Weissia* Probe” of species in the West Indies and adjacent areas radiating from the central core ancestral species *Neotrichostomum crispulum* (Bruch) R. H. Zander (Figure 6). This central species is modeled geometrically with four descendant lineages, and of these, two have four immediate descendants and one has three immediate descendants and one species of secondary ancestry. These models are neatly applicable in this particular group, but the number of descendants, immediate and secondary, is less clear in other groups, such as Streptotrichaceae [21]. The rule of four, however, is (to the extent studied) universal as graphed in Figure 1.

### 2.7. Model 7

**Figure 7:**
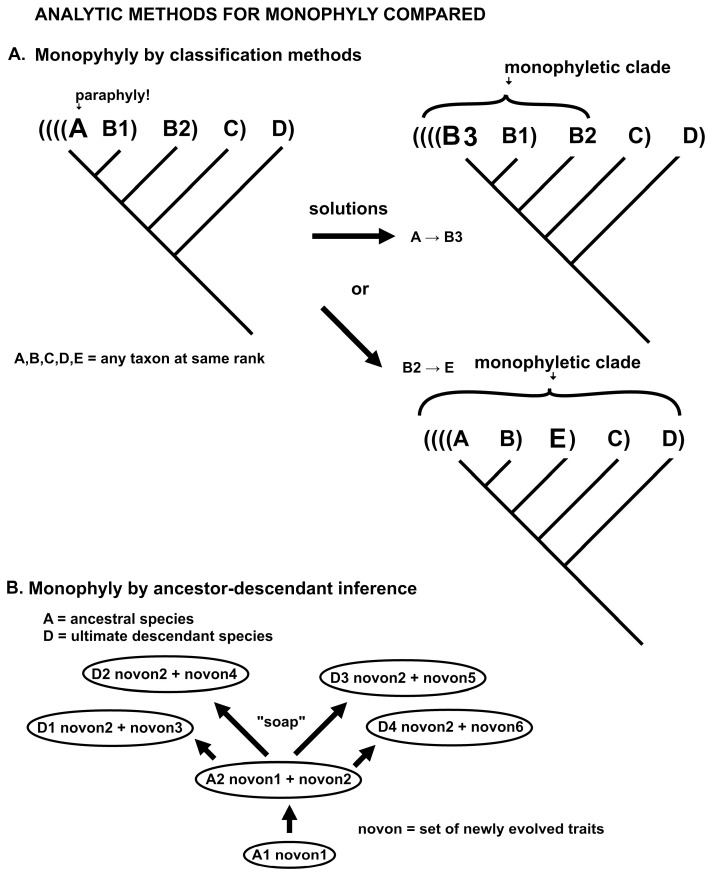
Comparison of analytic methods for determining monophyly. (**A**) Phylogenetics uses cluster analysis to estimate common ancestry and requires that by the Phylogenetic Classification Principle of Holophyly, all species in a cluster be of the same genus (or the same other rank). For a species to be a member of some phylogenetic genus, it must share the same ancestor as all the other species, that is, given the assumption that cladistically clustered species share the same ancestor, then to share an evolutionarily based name, they must share the same cluster. The poor solution ensuring overall monophyly is to either rename the unclusterable species to the same genus or to name all species as different genera. (**B**) High-resolution phylogenetics uses ancestor–descendant analysis to discover, among a larger monophyletic group, the internal branching train of minimally monophyletic genera. The key feature is the inheriting, to the descendant species, of the entire set of recent advanced morphological traits (novon 2) of the genus ancestor. This theoretically enables descendant survival peripatrically by species able to survive in two environments, a surfactant metaphorically labeled “soap”. The outgroup is species A1, the ancestor is A2, and the descendant species D1 through D4. For each descendant, “novon 2” is the set of new traits of the ancestor given to each descendant as a redundancy, and “novon 3”, “novon 4”, etc. are the new traits of each descendant.

The results mentioned above depend on a new approach to evolutionary analysis for classification purposes, that of ancestor–descendant analysis. The default method in systematics, and such as used in macroevolutionary studies in general now, is cladistics. A comparison of the methods is given in Figure 7. Figure 7A demonstrates that distinctive taxa embedded in large groups of the same rank are reduced to membership in that larger group. The form of monophyly employed by present-day phylogenetics is by the principle of holophyly, such that all species arising from the same ancestor must be at the same taxonomic rank, i.e., be in the same genus, family, etc. The ancestor is determined only as an unidentified shared ancestor by cluster analysis using advanced traits or probabilistic DNA base changes.

Ancestor–descendant analysis functions on the observation that an ancestral species gives each immediate descendant the same traits it developed as new when the ancestor itself originated through speciation. Novon 1 is the set of new traits of the penultimate ancestor. Novon 2 represents the new traits of the ancestor of all the descendants in its genus. It gives all immediate descendant species novon 2 entire, while each descendant also generate its own different novon 3 or 4 or 5 or 6, respectively. The interface of the immediate descendants with the ancestral species is, for purposes of argument, peripheral (peripatric) and involves novon 2 traits compatible in sympatry with the ancestor, and the other four novon trait sets adaptive to different allopatric environments. Thus, there is a speciational surfactant or “soap” in this model in part supporting the genus as a factual, complex, real, and universal evolutionary operator that is of taxonomic rank higher than that of species.

### 2.8. Model 8

**Figure 8:**
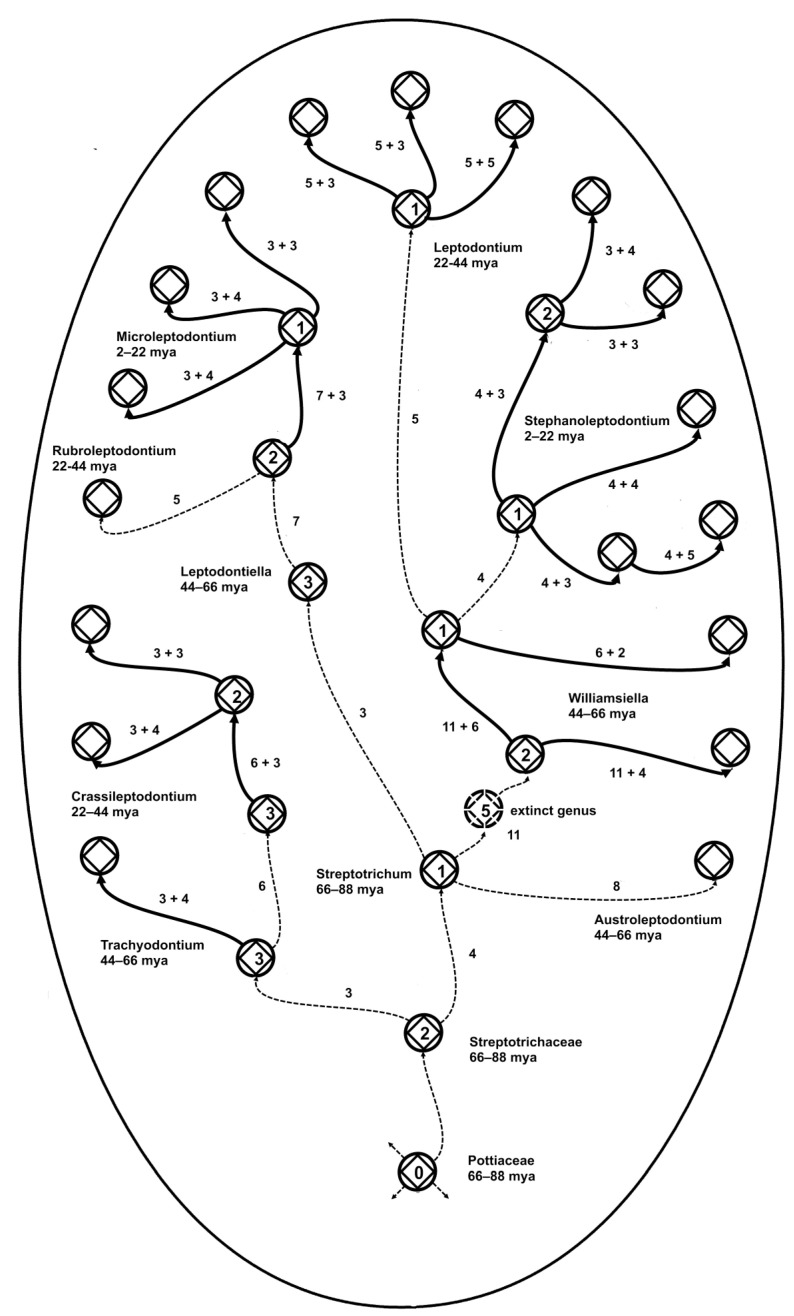
Example of the fractal surfactant in the moss family Streptotrichaceae, with estimates of age of origin. Species are represented by a presumed potential or actual ancestor (square on edge) and four peripheral potential or actual descendant species. Thick solid lines indicate ancestor–descendant relationships of actual species that possess the most recent new traits of the ancestor and the new traits of the descendant species; these are represented as a sum in bits (the minimum sequential Bayes posterior probability based on one bit per new trait). The dotted lines reflect evolutionary changes of the immediate ancestron between genera. Names of genera are given. One extinct genus is inserted based on a large (11 bits) evolutionary distance between two genera. Dotted lines are relationships between genera that only approximate the sharing of ancestral new traits with all descendants; bit numbers are new traits of their descendants. Numbers of extinct species may be estimated for genera by four minus numbers of actual descendants.

An evolutionary tree of stem taxa (caulogram) (Figure 8) for the moss family Streptotrichaceae [21,22] interprets the rule of four by showing the numbers of new traits (each set is a novon) associated with each speciation event. Thick lines track where sets of traits (the novons of the ancestor species in each genus) are shared by the ancestor and all of its descendants [16,22], except occasionally by secondarily derived descendants (descendants of descendants without descendants of their own). A spate of eleven traits between two ancestral species implies the extinction of an intermediate species, possibly the ancestor of an extinct genus. If each speciational constraint of about four descendants per genus and four trait changes per event is attributable to a power law generating process affecting evolution in general, then, this 88-million-year-old evolutionary tree [22] exemplifies the sturdy bones in the skeleton of evolution.

## 3. Discussion

### 3.1. Microgenera and Statistical Support

Analysis of morphological trait changes on a multichotomous tree with ancestral species assigned to the nodes allows tracing the actual pathways between minimally monophyletic groups that form the skeleton of larger monophyletic groups. Traditional phylogenetics, however, places all species, ancestral or descendant terminal on the dichotomous cladogram, even though Van Valen [49] pointed out in 1973 that ancestral species were both extant and common. A particularly cogent example of a coherent branching evolutionary tree of minimally monophyletic genera is that of the family Streptotrichaceae [21], with 10 genera and 26 species. Shannon–Turing sequential Bayesian analysis with second-order Markov chains allowed high minimum sequential Bayesian posterior probabilities with clear conjugate priors [16]. High-resolution analysis of monophyletic structure can be used to rescue apophyletic genera from epistemological extinction by cladistic synonymy [50] as is the case with the clade *Chionoloma* (=*Chionoloma* s.lat.).

### 3.2. Graphed Rule of Four

The evolutionary rule of four reflects the fact that four is the optimum number of new species generated by a single ancestral species (Figure 1), with variation doubtless due to extinction and secondary ancestry. There is also an average maximum of four new traits involved in speciation. The rule for traits is sufficiently stable that among the 10 genera in the moss family Streptotrichaceae, mostly different from each other by about four new traits per speciation, the 11 new-trait distance between two of the contiguous ancestral species implies the extinction of one species or more probably, given a great age of that portion of the lineage, an entire genus. There is evidence for the extinction of only one genus across an estimated 88 million years of existence of this family [22], implying that there may be lessons to be learned about evolutionary adaptive resilience in the face of environmental change.

Zander [22,35] showed that redundancy of apparently adaptive traits, similar to Shannon information optimization, is key to the survival of this family of mosses and perhaps any large taxonomic group. Hamming Code error correction commonly requires 1/7 of the total bits to be redundant. The redundancy of fractal microgenera is massive, with every immediate descendant carrying the most recently gained traits of the ancestral species. The error correction is most likely natural selection, through balancing selection plus extinction and replacement. For descendant species established allopatrically, redundancy becomes mutualism as novon traits of species sharing an environmental niche mesh ([19], pp. 118 and 121) to form another redundancy-supported group, the realized niche. How this, or other ingrained algorithms, may operate in nature requires much more research but should be of great value. Analysis of Shannon information and entropy in nature [51,52] is doubtless a guide.

Guy [45] lamented the fact that there are too few small integers for the multiplicity of processes involving simple relationships. Numbers with many numerals, including decimal fractions like pi or the square root of two, are unique and associated with particular processes in nature. As noted above in the discussion of balancing radiation, small whole numbers appear to be generated by a number of hidden processes that entail crowding or isolation. This is particularly true if associated with a power rule, or hollow curve, as illustrated in Figure 2 and Figure 6, which, at least, ensures the rarity of large numbers. Competition involves many traits, so descendant species as novel peripheral isolates may well be curtailed by complexity-based evolutionary processes restricting large numbers. Nature is apparently replete with processes governed or at least described by power rules and associated hollow curves implying fractal and self-similar results [44,53].

There are a number of rules of thumb regarding small whole numbers. The Rule of Five states that, with only five random samples from a population, there is a 0.9375 probability of having the median value between the smallest and the largest sample value [54], which is evidence of fractal and self-similar processes. Other rules are generally psychological. On the other hand, one explanation of Zipf’s law is a law of least effort [41], which restricts purposeful expenditure of negentropy, but, then, the similar physical law of least action [41] is still much of a puzzle (pace R. Feynman, who suggested that quantum effects cancel out all vectors but one).

Some cladists treat cladograms as real things that may be directly observed rather than inferred [55]. Clades form a nested hierarchy. A species may be paraphyletic (not monophyletic) if its definition excludes some morphological or molecular descendants, that is, if the ancestral population continues to be extant when some descendants are named a different taxon at the same rank. John Dewey ([56], p. 12) pointed out that “process”, as a universal, best embodied in modern natural science, is the “most revolutionary discovery yet made”. It replaces in philosophy such fixed and eternal absolutes as Being, Nature or the Universe, the Cosmos at large, Reality, or Truth. Dewey described a tension between those scholars with a fascination with sempiternal, immutable first principles and those with more pragmatic goals. This exists today, in my opinion, a similar tension with pattern cladists versus evolutionary taxonomists.

The actual difference between taxa and clades is that *taxa* are determined by trait changes between species modeling small bursts of speciation on a variously branching evolutionary model, while *clades* are determined by trait differences between clusters on a dichotomous dendrogram, modeling evolutionary splits (or coalescence) of clusters on an optimally fully resolved cladogram. Clusters do not evolve and their analysis may be of the cladogram branching order not necessarily inferred evolution [57]. The plausible evolutionary clustering of taxa on a cladogram is because evolution is such a powerful force that cluster analysis by any reasonable criterion results in plausible and apparently informative groupings. It is the fact that an enforced dichotomous tree cannot exhibit the distinctive patterns of taxa that the classification principle of holophyly is used to synonymize otherwise distinctive taxa into massive groups that are monophyletic in toto. The internal monophyly of minimally monophyletic groups (microgenera) is ignored as paraphyly.

The gradual replacement of taxon with clade, particularly in the well-regarded list of accepted taxa, like the cladist-curated World Flora Online [58], is also becoming common in vascular plant studies. The deprecation n in world-level online classification systems of many taxa, representing the results of a long and tedious expert study of units of evolution in nature, cripples the understanding of biodiversity. It is pointed out that the minimally monophyletic group, one ancestor and a few immediate descendants, is the basic unit of resilient evolution [5] and is a taxon, not a clade.

A suggestion for further study is that every process in nature that allows multiple elements to act as a unit leaves a power rule as evidence of the existence of that set of elements, in this case, taxa. Classical taxonomists have recognized these units, perhaps unknowingly, as demonstrated by the hollow curves in Figure 2. It takes taxon familiarity and expertise to deal with evolution involving trait changes at the species level, but the cladistic study is at least one step removed from the examination of the process of evolution.

This section deals with paraphyly and its destructive effects. See the discussion below for additional problems with cladistics analysis involving bad logic, modeling, sampling, and statistics.

### 3.3. Inscribed Polygons

Assuming speciation begins with relative isolation, consider a model [59] of such isolation, a circle representing the ancestral species. If immediate descendant species each having optimally four traits of the ancestor and four new traits are to survive at the periphery of the ancestor’s range (geographic, habitat-wise, or event time), and survive competition with each other, then a geometric model (Figure 3 and Figure 4) minimizing competition might be a regular polygon inscribed in a circle. The areas between the polygon and the outer limits of the circle are isolated from each other and about equal in size. Of course, a peripheral area with only one or two descendant species offers them abundant room for survival, but the number of additional descendant species is limited by a rapid decrease in the areas allotted by inscribed regular polygons of a larger number of sides (see Figure 6).

The maximum area for selective adaptation and survival of the descendant species, inferred from extant data on species per genus, is anywhere from 1 through 5 species on a hollow curve depicting the ratios of areas cut off by the regular polygons to the area of the full circle, as illustrated (Figure 3). Equation (1) gives the formula for the ratio of the area outside the inscribed polygon to the area of the circumscribing circle.

The formulae dealt with here have exponents of minus one or a fraction of one. What about models in three dimensions? There are five regular Platonic solids: tetrahedron of 4 sides, cube of 6 sides, octahedron of 8 sides, dodecahedron of 12 sides, and icosahedron of 20 sides. Each solid will fit inside a sphere and also touch the surface with its vertices. Each of the volumes between the sides of the solids and the sphere surface will be equal, but the volumes are exposed to each other contiguously. This exposure is analogous to competition and would be considerable, since the proper model requires little or no competition. Competition is reflected in Turing patterns [47,48], which are the results of competition between internal chemical processes in nature, including in a living body. Examples are the stripes on a tiger. If the geometric patterns modeled in Figure 3 and Figure 4 are extended through time, then the striations representing peripatric ranges of descendants are direct results of competition similar to those of Turing patterning and may be governed by apposite equations.

### 3.4. Peripatric Competition, Balanced Radiation, and Punctuated Equilibrium

A balanced radiation of one or two descendants may occupy up to half the area potentially held by the ancestral species. Regular polygons inscribed in a circle demonstrate rapid loss of potential habitat for increasing balanced numbers of descendant species occupying peripatric habitats without overlap with other descendant species.

More than five descendant species are liable to extinction due to the small size of available survival space (habitat, competition load, lack of mutualism). This model of balancing radiation provides a causal explanation for the fractal nature of evolution, where four products are added to the generator to obtain the ratio 5:4, or a fractal dimension of 1.16 (log 5 divided by log 4), equivalent to the well-known Pareto distribution of 80:20.

This is the model with balanced distribution around a circle, but what about the periphery of a sphere? Externally, only two regular polygons can completely cover a sphere, viz. triangles and squares. Any genus then may generate very few descendant species that balance each other in mutualism and competition. The above formulae do not involve squares (or cubes) and seem sufficient, however, to describe the effects of evolutionary processes in nature. Pentagons and regular polygons of more sides require additional, different polygons to completely cover a sphere. Penrose tilings can cover a sphere in an approximate manner, but the areas of the two main tiles continue to approach the Golden Ratio of 1.618; that is, they remain unequal.

One might note that the soap analogy (Figure 8) of new species in a balance of about half sympatric and half allopatric might apply to the realized niche, that is, the actual species in the somewhat slippery concept of a niche. The first colonizing species determines the nature of the survival traits and subsequent colonizers of different species match up their newly evolved traits while competing peripherally with geometric limitations on the competition for size of survival areas. This assumes all species involved are about as similar as the descendant species of one ancestral species; that is, a shrub is not part of a geometrically affected realized niche of several mosses. No data are as yet available for this analogic hypothesis.

### 3.5. Sheaves of Hollow Curves

Numbers associated with statistics can be confusing. For an unimodal binary distribution (a bell-shaped curve), 30 samples are commonly considered sufficient to characterize that curve at 0.95 probability. This is because 95/100 is equivalent to 19/20, and information about the 1/19 of the distribution may be had with confidence if the numbers of samples were increased half-again from 20 to 30. This gives confidence in the known variability of the species.

A Shannon information bit represents halving the uncertainty. Each decision of which direction a trait has changed in evolution (i.e., as information about which species is ancestor and which is descendant) is assigned one Shannon informational bit. Equation (4) shows bits *h*(*x*) equal to the logarithm at base 2 of the inverse probability of *x*, that is, of *P*(*x*). Equation (5) demonstrates that for two alternative outcomes (two different trait change directions or probability 1/2), a decision on one of them yields one informational bit. Bits are additive and an odds table [16] gives the Bayesian posterior probability that some one species is the descendant of the next.

(4) $$h\left(x\right)={log}_{2}\frac{1}{P\left(x\right)}=-{log}_{2}P(x)$$

(5) $$h\left(x\right)=-{log}_{2}\frac{1}{2}={log}_{2}2=1$$

This method of determining support is Bayesian because the statistical analysis is (1) a second-order Markov chain, with support for the putative ancestor coming from both the matched primitiveness with the outgroup and it being more generalist than advanced traits of other species in the genus, and (2) it is sequential Bayes in which all bit assignments in a series may be added to derive a very high support measure for any length of concatenation in an evolutionary tree (i.e., as high an order of the Markov chain as there are n − 1 species, support being both backwards and forwards on the evolutionary dendrogram).

Five is an important figure in statistics because (1) bit distribution 1.0 to 4.3 (average) spans the first and second standard deviation of variation [60] and (2) the “rule of five”, a rule of thumb which states that a random sample of five from a population will include, between the smallest and largest value, the medial (center value) of that population at 0.9375 probability [54]. The upshot is that four descendant species, each with four new traits, have an excellent chance of efficiently addressing most of the contiguous allopatric habitat with adaptive traits. More descendant species and more new traits per speciation event may be superfluous and a burden on available survival-critical biomass and energy. Speculative? No. Empirical evidence for this explanation is present in the hollow curves of species per genus (Figure 2) that are theoretically due to natural selection, while the worked-out evolutionary skeletons are solidly articulated at the nodes. More work at this level is, of course, called for.

The empirical distribution of the evolutionary rule of four (four new descendants, four new traits) may be interpreted as the dispersal of information. For a point source or stationary circle, measures of increases in information will involve unitary or fractional exponents. When a radius is increased, the circle enlarges and creates new information as a numeric square. The circumference of a circle inscribed or generated on a spherical surface increases with the radius halfway around, then decreases to a point on the other side. A saddle-shaped surface with frilly edges has the circumference increasing more rapidly as the radius increases. Areas associated with the periphery of the circumference increase with the square. Additional factors that affect the rate and interspersal of peripatric generation and competition of descendant species include fragmentation by continents and islands, differential extinction, and serial compression by global glaciations. The devil is in the biasing context as we create information on geometric models as practical data sets relevant to the analysis of natural processes. The rule of four signal seems to come through, however, both in the small (Figure 1) and in the large (Figure 2).

Using trendlines in MS Excel allowed the characterization of the curves in Figure 5. The curve for Zipf’s law is clearly a power rule, matching the Excel trendline for power. The other hollow curves are exponential for higher values on the *y*-axis with values near one but follow a power law in the tail, which together is apparently common in empirically derived curves found by “best fit”.

The close association of the hollow curves in Figure 5 may be interpreted in different ways. The curves could be each an approximation of a single basic curve that rules them all, or they could be each valid and accurate descriptions of the results of several closely related processes in nature. The second explanation is attractive and fits the modern recognition that there may be different but equally valid explanations for similar natural phenomena. This is why one searches for a best-fitting curve rather than some obvious single solution. The hollow curves are integrated summaries of how the rule of four has constrained minimally monophyletic units over 88 million years of biogeographical modification of the modern flora. Certainly, there are different rules for observed features of the quantum microcosm, Newtonian mesocosm, and Einsteinian macrocosm, while wave and particle observations are problematically explained as a single process, and the Theory of Everything remains obdurately hidden.

It is suggested that if there is a single formula that explains evolutionary information adequately, it will involve wrinkles in informational space-time. By this, the author means that the informational context affects the description of the results of the process in nature. Just as inscribing a circle on a sphere or on a negatively curved surface (a saddle) must modify the exponent (assuming no change in the constant π), the geometric models may suffer from the confounding effects of inconstant reality. For this reason, it is suggested that the sheaf of curves in Figure 6 reflects a conclusion that evolution is quasi-Zipfian and describable only with Zipf-similar formulae, including the meta-law formula of Constantin et al. [42].

Power law and exponential descriptions of the results of processes in nature may be traced to one feature, the dispersal of created information with increasing radius from the center. Speciation is a perfect example of the creation of information around the periphery of an originating source, with increasing information, to get an exponent of minus one if information increases with no increase in radius, 2 if area, 3 if volume, 4 if an additional dimension is considered. All variables that are needed to give the best fit to the data reflect the different contexts in nature or wrinkles in informational space-time. The several hollow curves, both exponential and power law (depending on Excel trendline matches), are the informational bones of the skeleton of evolution, and all explain or at least reflect, variously, the rule of four in speciation.

### 3.6. Actual Evolutionary Tree and the Rule of Four

An evolutionary tree (Figure 6) of a set of closely related species in the West Indies and adjacent mainland was inferred in a previous paper [19]. This tree is presented here with genera modeled as geometrical shapes with descendant species as areas outside polygons inscribed in circles. The rule of four is clearly evident in this diagram and, in fact, also evident at the supra-generic level (as self-similarity) with four descendant genera from *Neotrichostomum crispulum.* It is suggested that perceived suggestions or indications of such branching gave classical taxonomists in the past the reason to describe so many small genera (Figure 2). Two of the lineages are mostly local in provenance and are inclusively coherent; the remainder extend their lineages to other parts of the world and involve other species.

### 3.7. Methods of Evolutionary Analysis and Classification

Critiques of scientific methods are generally ineffective unless an alternative, better method is offered. Such an alternative, macroevolutionary systematics, has been described in detail in several previous publications. It is a new science but, like traditional cladistics, is based on numerical taxonomy. Essentially, it is high-resolution phylogenetics. Phylogenetics may be defined as studying the evolutionary relationships of organisms with numerical techniques, particularly of statistical support for dendrogram branches, reflecting its origin in computational systematics. Cladistics is the classification of organisms by shared derived traits, implying common ancestry of clusters. Phylogenetics is commonly identified with cladistics; however, there are other methods of inferring evolutionary relationships in a computational context. High-resolution phylogenetics infers relationships of organisms by ancestor–descendant trait changes between species, emphasizing descent with modification and focusing on connections between minimally monophyletic groups.

The protocol, in short, is as follows: Morpho-species are segregated into small, closely related groups using any standard hierarchical method, limiting the relative data sets so as to minimize the effect of convergent traits. Genera are identified as (1) minimally monophyletic groups with one ancestral species that is most similar to the outgroup and also least generalist among the ingroup; (2) the newest traits of the ancestral species are found reproduced in each of the descendant species; (3) there are usually up to four immediate descendant species in each genus and about four new traits in each species; and (4) a maximally parsimonious (maximum informational entropy) dendrogram is produced, which is a multichotomous tree with extant or inferred ancestral species at the nodes (a caulogram). Statistical support is through Turing–Shannon sequential Bayes using one bit per new trait on a second-order Markov chain.

The branching concatenation of ancestral species provides a monophyletic evolutionary skeleton to larger monophyletic groups. Confirmation of the morphological skeleton is presently by identification of paraphyletic species on molecular cladograms, which are presumed to be ancestral to other species. This has been proven true in a study of the moss clade *Chiololoma* [5] (Zander 2024e). One may hope that future molecular analysis using caulograms will provide more information. Mapping of morphological traits to molecular cladograms is now unnecessary.

This recasting is a major fix for the traditional methods of cladistic systematics, which is a 50-year-old technology. Originally, a cladogram was described as simply a branching network of ancestor–descendant relationships ([61], p. 28). Cladistics presently focuses only on the estimated order of branching not the order of taxa, and the former supposedly identifies common ancestry. It rejects ancestor–descendant analysis by suppressing information from any paraphyly, morphological or molecular. Hörandl [62] has pointed out that commonly used sequence markers are limited to grouping evolutionarily, while expressed morphological traits that contribute to structure and function are deeply involved in selection, adaptation, and co-evolution and, thus, may be the proper bases for evolutionary grouping in classification. The classical morphological taxonomic method is based on hard-won informal genetic algorithms resulting in well-tested heuristics commonly known as expertise [63,64].

It is uncomfortable to suggest that cladistics is fundamentally anti-evolution [57]. The PhyloCode [65] glossary defines “phylogenetic” as of or pertaining to the history of ancestry and descent. Abundant evidence in cladistic publications, however, suggests that cladism suppresses any suggestion of descent with modification, that is, of ancestor–descendant transitions between extant species, although lip service is paid to “common ancestry”. The fact is that evolution, either gradual or by short jumps of a few traits per speciation event, has so powerful an effect for grouping by similarity that any cluster analysis by any reasonable criteria will generate evolutionarily somewhat coherent groups. Without close analysis of ancestor–descendant changes at the species level, both morphological and molecular, cladistic study results in poor resolution of monophyly and large genera as clades bloated with holophyletic synonymy. Evidence that cladistics rejects or strongly minimizes evidence of descent with modification includes the following, to a great extent corrected in the new high-resolution phylogenetics:

1. A cladogram, as an enforcedly dichotomous dendrogram, cannot exhibit the distinctive multichotomous patterns of the little ancestor–descendant bursts of speciation characteristic of evolution. A cladogram originated as a visual aid for sorting out the nested parentheses of cluster analysis.
2. A cladogram does not have extant taxa assigned to an internal node, even though about half of all species are ancestral to other species. Determining common ancestry of bursts of one to four descendant species as pairs of taxa is fraught.
3. Phylogenetic analysis is restricted to a narrow form of eliminative induction, asking the question: “which cladogram best explains the data?” not “which of any reasonable tree is optimal?” The “black box” of massively incomprehensible statistics (to the average taxonomist) obscures this essential bias.
4. The terms ancestor and descendant have been replaced with “paraphyletic” and “apophyletic”, respectively. These latter terms describe the positions of nodes on the cladogram, not evolution.
5. Analysis with a first-order Markov chain on a dichotomous dendrogram selects optimal sister groups and that with a second-order Markov chain on a multichotomous dendrogram selects optimal ancestor–descendant sets.
6. Clades have little value in floristic studies. A clade consisting of a paraphyletic species or genus plus all descendant species or genera has no or a much diluted evolutionary trajectory, characteristic ecology, or value in biodiversity study.
7. A large number of randomly sampled exemplars of a species can reveal the molecular paraphyly to identify an extant ancestral species. Looking for paraphyly as evidence of descent with modification is not a goal; however, even though funds for adequate sampling are abundant.
8. Rather than follow the PhyloCode [65], clades are named as taxa although clades are not taxa. A *taxon* is a set of organisms distinguished by expert evolutionary taxonomists intimately familiar with the group, its variation, and ecological features. A taxon consists of organisms very similar in overall homologous traits, generally evinces a morphometric gap or distance, and seems to have a unique evolutionary trajectory. As presented here, a genus is a fundamental unit of resilient evolution [5], defined as a minimal monophyletic group of one ancestral species and a few (ca. 1–4 immediate descendants) and exhibiting a rule of four (optimally four descendant species, all species with mostly four new traits). Given the fractal nature of the genus, self-similarity is evident at all other taxonomic ranks, species, and families [19] (Zander 2023). A species is a set of organisms describable by any of the usual definitions of species as long as such species can participate in evolutionary lineages in a minimally monophyletic genus.

A *clade* is defined in the PhyloCode [65] Art. 2 as “an ancestor (an organism, population, or species) and all of its descendants”. A clade differs from a taxon in its modification to follow the classification principle of holophyly, usually in practice of a paraphyletic species, genus, or family being merged with any apophyletic (descendant) taxa of the same rank. A taxon is an evolutionary unit, a clade is commonly a composite of more than one evolutionarily distinctive taxa. The moss family Pottiaceae Hampe is now a clade [58] in the influential World Flora Online having been merged, because of paraphyly, with descendant families Cinclidotaceae Schimp., Ephemeraceae J. W. Griff. and Henfr., Splachnobryaceae A. K. Kop., Streptotrichaceae R. H. Zander. The pottiaceous genus *Syntrichia* Brid. is now a clade because it has been stuffed with the otherwise well-established taxa *Calyptopogon* (Mitt.) Broth., *Dolotortula* R. H. Zander, *Sagenotortula* R. H. Zander, *Streptopogon* Wilson ex Mitt., and *Willia* Müll. Hal., and all their species were transferred [66] to combinations in *Syntrichia*. There are many other examples of clades presented with taxon names.

The Phylocode recommends (Rec. 6.1B) that the letter “P”, bracketed or in superscript, be used to designate clade names and the letter R used for rank-based names. Thus, the taxon Pottiaceae, when appropriate, would be addressed as Pottiaceae [R] but as a clade, it would be Pottiaceae [P]. If treated as both a clade and a family in the same publication, they should be clade Pottiaceae versus family Pottiaceae. The author has not seen this done, and clades abound in the taxonomic literature that are not identified as such. The difference in biotic significance may be immense between a clade named as family, genus, or species and a taxon named as family, genus, or species.

### 3.8. Caulogram of Streptotrichaceae

The evolutionary tree of the moss family Streptotrichaceae (Figure 8) is based on ancestor–descendant analysis using morphology; there is no molecular work available to search for evolutionarily informative paraphyly. It is developed by second-order Markov chain analysis, in which the ancestral species for each genus is identified as most similar to the outgroup or nearest genus and most generalist among the ingroup (remainder of the species in the genus). It shows an evolutionary skeleton of connected ancestral species of minimally monophyletic genera embedded in a larger monophyletic field, the family. Names at any rank are of taxa.

Statistical support is through Shannon–Turing sequential Bayesian calculation, where each new trait is assigned one informational bit, and these are added across speciation events and interpreted with an odds table for posterior probabilities. Conjugate priors are fully expected [16], with probabilities for and against most probable genus patterns adding to 1.00. Homologous morphological traits are used, which in most cases give unambiguous transitions of state changes. This leads to accurate identification of “surfactant” traits (small sets adaptive in two different habitats) suggested as key to survival peripatrically and of novel traits in descendant species apparently always sourced as state changes from older, apparently unused traits of the ancestral species. The conservation of the advanced traits of the ancestral species among the descendant species implies a kind of punctuated equilibrium preserving the genera as a whole over time. Few genera have extinct ancestors (which are inferred by their intermediate traits). A parsimony of state changes with apparently successful traits “banked” or preserved in the genomes of coeval descendants is suggested as key to the long-term survival over 88 million years for this medium-sized family of mosses [35].

## 4. Materials and Methods

The regularization of proportionate generation of numbers of descendants and morphological traits (i.e., a catchall term for all genetic expressions) were investigated: Numbers of species per genus and average numbers of new traits per species per genus are graphed (Figure 1). Comparison was made of the numbers of species per genus with areas between ranked regular polygons and the circles in which they were inscribed (Figure 1 and Figure 2). Comparison also was made of ranked circles with inscribed regular polygons or their equivalents as models of peripatric speciation and minimized completion between coeval descendants (Figure 3). Estimates were made of changes in peripherally generated new species over time (Figure 4).

Comparison was made between the geometric rule of ranked inscribed regular polygons with Zipf’s law and with the Constantin et al. meta-law [42] in physics (Figure 5). Modeling ([8], p. 354) was performed on an actual macroevolutionary pattern in nature with geometric interpretation (Figure 6). The comparison was made of the geometric interpretation of monophyly in ancestor–descendant systematic analysis and phylogenetic analysis of monophyly (Figure 7). An evolutionary tree of stem taxa, i.e., a caulogram, was adapted from a previous taxonomic study of the moss family Streptotrichaceae [21,22] to demonstrate numbers of extant descendant species per genus and numbers of new traits (the novon set) generated per speciation event. Thick lines on this tree show connections between species that share identical sets of the newest traits of the ancestor, apparently facilitating survival in peripatric areas (Figure 8).

The calculations, graphs, and formulae were generated using a Microsoft Excel 2016 spreadsheet, with figures composed on PixelNeo Image Editing Software, ver. 2018-8-28. Voucher specimens examined in research leading to this study are listed in the relevant publications cited here.

## 5. Conclusions

The geometric model of peripatric speciation is based on the inference of biological lineages as multichotomous with one ancestral species giving rise to optimally four immediate descendants. Trait changes occur between species, not clades. Evolution and taxa are real things in nature [67,68] to the extent that these phenomena are best modeled and tested. The hollow curves of species per genus in older taxonomy mean that the effects of process-based power laws were evident even in “intuitive” methodologies of the past.

Much information about common ancestry in evolutionary relationships on a grand, macroevolutionary scale is now being presented in cladistics-based phylogenetic or phylogenomic analyses. Traditional cladistic methodology is, however, inaccurate and destructive. Both ancestral and descendant species are clustered by molecular or morphological traits or both, on dichotomous trees or cladograms, which is a constraint that does not reflect a process in nature. Cladograms summarize steps in cluster analysis by advanced traits or maximum probabilities of DNA base transitions and are not evolutionary trees. They are essentially visual aids for understanding the relationships of clustered taxon names that are often deeply nested in sets of parentheses as Venn diagrams (see Figure 7).

Cladistic analysis stuffs data on a naturally multichotomous tree of evolution into a dichotomous dendrogram. The result is that natural taxa are stripped of distinctive patterns. Because genera and species have no characteristic patterns possible on a fully resolved dichotomous tree, these taxa can have no cladistic presence. To make up for this, the concept of a clade was introduced, being simply a set of all organisms descendant from one ancestor, which led to the principle of holophyly—somewhat paradoxically that descendant taxa cannot be named at the same rank as that of the ancestral taxon, e.g., no species can descend (in name at least) from a species, ditto for genus, and for family. The idea of descent with modification, then, given the inadequacies of the dichotomous tree, was abandoned in favor of common ancestry.

Actual molecular evidence of ancestry is the nesting of one taxon in another of the same rank, commonly dismissed as paraphyly. The nested taxon is apophyletic, and the nesting taxon is made paraphyletic by the apophyletic taxon because the paraphyletic taxon does not entirely share the same shared cladistic ancestor. In traditional cladistic phylogenetics, if genera are nested, then they are synonymized; if species, then ditto; if specimens, then the nesting specimens may be named as new cryptic species or all specimens renamed as the same species (Figure 7). Multichotomies in a cladogram are commonly dismissed as “unresolved”, with little information in them because represent two equally probable trees. The solution is clear, to use a multichotomous tree with putative ancestral species at the nodes, and abandon cluster analysis in favor of ancestor–descendant analysis. Essentially, clades are artifacts of the low resolution of dichotomous trees with unnamed nodes. High-resolution phylogenetics can with facility replace clades with taxa. The requirement, of course, is the expertise of classical systematics.

A species that has a molecular variant as a sister group and another variant of that same species as the nearest neighbor is also vulnerable to non-acceptance as a named entity. This practice is thankfully not yet prevalent. It is surprising, for example, that *Chionoloma dubium* (Thér) M. Alonso, M. J. Cano, and J. A. Jiménez is not synonymized with *C. angustata* (Mitt.) M. Menzel, inasmuch as the former is clearly apophyletic with three sets of nearest-neighbor clades of the latter, molecularly paraphyletic species [69,70]; these species are quite distinct morphologically, which may account for the authors’ reserve. Remember that for every four immediate descendant species, there is, possibly extant, a cladistically confounding molecular variant of the ancestral species with the same molecular signature as its descendant species.

Paraphyly is valuable evidence of the descent of the apophyletic taxon with modification. All paraphyly on a molecular cladogram is an ancestral signature, but because of inaccurate clustering on a dichotomous tree, such information to be effectively used must be corroborated by morphological or molecular ancestor–descendant analysis on a multichotomous second-order Markov chain, which, if molecular, should use informative priors with evolution tracked probabilistically by DNA sequences relatively unaffected by natural selection (i.e., by convergence).

The adoption of morphology-based macroevolutionary systematics is here advocated because it is better science than either cladistic morphological or molecular phylogenetics. It is better science because (1) genera are empirically defined; (2) the multichotomous tree patterns match the inferred generation of descendant species; (3) there is a clear evolutionary path between genus ancestors that stitches together minimally monophyletic groups of one ancestral species and a few descendant species; (4) statistical support by Shannon–Turing sequential Bayes involves true Bayesian priors and conjugate priors and guides tree-building; (5) the Markov chain generates more information in being second order and multichotomous; (6) the model demonstrates an internal, fully monophyletic skeleton within a larger monophyletic assemblage; and (7) the model allows description and prediction of resilience of large groups over vast time frames.

In addition, morphology deals with just a few straight-forward expressed traits that facilitate evolution per speciation event, while molecular studies introduce uncertainties including alignment, wrong gap costs, differential lineage sorting, hybridization, polyploidy, recombination, non-clocklike behavior, rates other than gamma distributed, differences between the results of “total evidence” and evaluations based on separate gene studies, possible strong selection pressure on non-coding promoter sequences, persistent pseudogenes, too few exemplars, endogenous retroviruses, gene conversion, self-correction of flawed DNA, paralogy, codon bias, chloroplast capture and other horizontal gene flow, novel clades, saturation, third codon bias, wrong identifications, long-branch attraction, and model insufficiency; among others, even rare cases of molecular convergence between unrelated taxa [71].

The supreme result of taxonomic experience over the past 250 years is the generation of a large, predictive model of the evolution of biodiversity, and it is being dismantled one synonymized genus at a time through ad hoc principles associated with short-cuts to science. The tools of phylogenetics are blind to the internal skeleton of minimally monophyletic genera in large taxonomic groups. The central, supportive structure of concatenated minimally monophyletic genera, however, through fractal trait redundancy controls and potentiates survival of the supra-generic group through the 66 million years of existence of the modern flora [35]. Evidence is thus available that evolution, as descent with modification, may act in massive survival force at the genus level, while the present literature on species competition is akin to local skirmishing. This is new science.

## 6. Summary

There has been an unacknowledged battle going on since the early 1970s, of numerical taxonomy against classical taxonomy, which is now deprecated as “traditional” or “intuitive” analysis. Essentially, this fight is of cladistic common ancestry versus evolutionary descent with modification. Although both can reveal evolutionary information, the former is gradually eroding the field of taxonomy by suppressing information about descent with modification (deprecated as “paraphyly”) and taxa as products of evolutionary processes (because distinctive taxon patterns cannot be shown on an enforced-dichotomous cladogram). Common ancestry is shown by character state changes from cluster to cluster, not a model of evolution, while descent with modification is demonstrated by character state changes from ancestral species to descendant species, which fits evolutionary theory. Herbaria have begun to organize their collections by clades labeled with “traditional” names, but clades and taxa are different sets, one fitting a cladogram, the other fitting evolutionary theory. Apophyletic species are also vulnerable to epistemic extinction. Molecular phylogenetic systematics and its associated classification principle of holophyly are gradually eroding taxonomy to create an evolution-free and taxon-free knowledge tree structured on a cladogram. Dealing with the biodiversity crisis will suffer without modeling evolution as descent with modification or having taxa as conceptual handles of processes in nature. The author has proposed a scientifically and statistically rigorous protocol for modeling and classifying biodiversity evolution with high-resolution analysis of monophyly, that is, explication of structural monophyly of large monophyletic groups through identifying their included minimally monophyletic groups.

## Figures and Tables

**Table 1:** Comparison of the area of a circle outside an inscribed regular polygon with physics meta-law and Zipf’s power law series corresponding to the number of polygon sides as ranked data. Polygons of sides 1 and 2 are conjectural, estimated by regression, but represent steps in speciation by balanced radiation with minimum competition between descendant species.

Number of Polygon Sides	Maximum Fraction of Circle Excluded	Zipf’s Law	Physics Meta-Law
1	0.67	1.00	0.72
2	0.50	0.50	0.51
3	0.37	0.33	0.37
4	0.27	0.25	0.26
5	0.20	0.20	0.19
6	0.15	0.17	0.14
7	0.11	0.14	0.10
8	0.09	0.13	0.07
9	0.07	0.11	0.05
10	0.06	0.10	0.04
11	0.05	0.09	0.03
12	0.04	0.08	0.02

## Data Availability

All data are provided in the present manuscript.

## Glossary

Ancestron	All the morphological traits of the ancestral species.
Apophyly	A cladistic term for the position on a cladogram of a descendant taxon. For the cladistic term “embedded”, read “descended from”.
Caulogram	A multichotomous tree modeling evolutionary patterns with nodes assigned taxon names.
Cladistics	Cluster analysis by parsimonious trait changes or probabilistic base pair changes. Results are displayed by a cladogram.
Cladogram	A dichotomous tree, originally intended as a visual aid to interpreting results of cluster analysis as the often deeply nested parenthetical names placed terminally on the tree.
Epistemic extinction	The case when taxa determined as evolutionary facts are synonymized with earlier names by the cladistic principle of holophyly such that they disappear thereafter from scientific discourse, e.g., floristic lists.
Genus	A minimally monophyletic group with mostly four immediate descendant species and mostly four traits per species, with its own new traits transferred to each immediate descendant. The fractal dimension of a genus of five species, four of which are descendant species, is $\frac{\log5}{\log4}$ or 1.16, apparently universal, and self-similar to the observed patterns in the next higher rank, the family (or supergenus, subfamily).
Immediate ancestron	The traits preserved redundantly in a minimally monophyletic genus. The set of traits that are newly evolved in the ancestral species and which are given entirely to each immediate descendant species.
Minimally monophyletic genus	An evolutionarily active group of one ancestral species, extant or inferred, and a few immediate descendants. Optimally, there are four immediate descendant species; also termed a microgenus or monothetic genus in other papers.
Minimized competition between coeval descendants	Maximal survival of a genus is supported by about even-sized areas of immediate descendant species with minimal inter-descendant competition and maximal survival of the redundant immediate ancestron traits.
Morphology	A catch-all term for all the expressed traits of a taxon.
Novon	The set of new traits of descendant species obtained by trait changes of less-useful older traits of the ancestral species. The number of such traits is commonly two to seven, but most generally only four.
Paraphyly	The position of a taxon on a cladogram indicating it is ancestral. Usually a sister species with a nearest neighbor of the same name and taxon rank.
Peripatric speciation	The idea that speciation most probably occurred at the margins of the distribution of an ancestral species.
Phylogenetic analysis	Using numerical methods to determine monophyly. The standard method is cladistics, which is destructive and inaccurate; destructive in classification because its low resolution requires holophyletic synonymizing of taxa, each previously determined as evolutionary units, into large clades that name overly broad evolutionary units, and inaccurate in ignoring descent with modification of molecular variants of the ancestor that match each descendant. High-resolution phylogenetics uses Shannon–Turing analysis on second- or even multi-order Markov chains, with taxa investigated at nodes of multichotomous trees.
Power law	The hollow curves representing species per genus and genera per family exemplify logarithmic distributions, rising high on the left and extending down monotonically to the right. A power law is a distribution that makes a straight line graph with both x- and y-axes scaled logarithmically. Visually, the hollow curve has a longer and broader asymptote on the *x*-axis than an exponential curve.
Rule of four	There are commonly four immediate descendant species and four novon (newly established) traits in a minimally monophyletic genus. From parallel features reported in inorganic chemistry.
Second-order Markov chain	Bayesian posterior probabilities can be calculated by adding Shannon bit probabilities of outgroup to ancestor as prior, and of ancestor to descendant as likelihood. A multichotomous Markov chain (tree) node is then determined by two sets of morphological data, not just one as in first-order Markov changes used in molecular systematics.
Secondary ancestry	When a descendant species generates another descendant species or two, but these not being genera themselves.
Self-similarity	A feature of complexity theory in which the same structure or process is repeated at different scales, e.g., Mandelbrot designs.
Shannon information theory	Given only two directions for an ancestor–descendant trait change, a decision one way or another yields 1 bit, where a bit is defined as—log_2_ ½, which is log_2_ 2, which is 1. One bit decreases uncertainty by one-half. In Turing sequential analysis, bits as trait changes from ancestor to descendant can be added because they are logarithmic, one bit per trait change. Probabilities are read from an odds table for sums of bits from ancestor to descendant or any length of the lineage.
Speciational surfactant	In immediate descendants in a minimally monophyletic genus, the set of new traits in the ancestral species (the immediate ancestron, usually four traits)—which enable differential survival for that ancestral species—almost always together with a set of newly evolved descendant traits (the novon, usually four) in a descendant species. Then, one can analogize a chemical surfactant (“soap”) with molecules soluble in an ionic liquid (allopatry) and also in a nonionic liquid (sympatry) at the margins of an ancestral range (peripatry). This is a feature of evolution hypothesized as balancing survival potential for the genus as a whole.
Taxon	A group of organisms determined by a taxonomist as having some evolutionary coherence and connected with related groups by common ancestry and descent with modification.
Turing patterns	Certain distinctive patterns occurring in nature, e.g., zebra stripes, leopard spots, and distinctive distribution of plants, were hypothesized by A. Turing as due to competing biochemical processes. Laboratory experiments have duplicated this phenomenon.
Turing sequential Bayesian analysis	The use of the Bayesian posterior probability as the prior of the next computations using Bayes’ formula, when additional data become available.
